# Thyroidal Transcriptomic Profiles of Pathoadaptive Responses to Congenital Hypothyroidism in XB130 Knockout Mice

**DOI:** 10.3390/cells11060975

**Published:** 2022-03-12

**Authors:** Junichi Sugihara, Aaron Wong, Hiroki Shimizu, Jinbo Zhao, Hae-Ra Cho, Yingchun Wang, Samuel Refetoff, Peter Arvan, Mingyao Liu

**Affiliations:** 1 Latner Thoracic Surgery Research Laboratories, Toronto General Hospital Research Institute, University Health Network, Toronto, ON M5G 1L7, Canada; jsugihar@g.ucla.edu (J.S.); aaron.wong@uhnresearch.ca (A.W.); hiro0810@koto.kpu-m.ac.jp (H.S.); zhaojinb@fmmu.edu.cn (J.Z.); haera.cho@mail.utoronto.ca (H.-R.C.); yingchun.wang@utoronto.ca (Y.W.); 2 Institute of Medical Science, Temerty Faculty of Medicine, University of Toronto, Toronto, ON M5S 1A8, Canada; 3 Department of Physiology, Temerty Faculty of Medicine, University of Toronto, Toronto, ON M5S 1A8, Canada; 4 Departments of Medicine, Pediatrics and Committee on Genetics, The University of Chicago, Chicago, IL 60637, USA; srefetof@uchicago.edu; 5 Division of Metabolism, Endocrinology & Diabetes, University of Michigan, Ann Arbor, MI 48105, USA; parvan@umich.edu; 6 Departments of Surgery and Medicine, Temerty Faculty of Medicine, University of Toronto, Toronto, ON M5S 1A8, Canada

**Keywords:** thyroid hormones, goiter, gene set enrichment analysis, mitochondrial energetics, extracellular matrix, inflammatory response

## Abstract

Congenital hypothyroidism is a genetic condition in which the thyroid gland fails to produce sufficient thyroid hormone (TH), resulting in metabolic dysfunction and growth retardation. Xb130^−/−^ mice exhibit perturbations of thyrocyte cytoskeleton and polarity, and develop postnatal transient growth retardation due to congenital hypothyroidism, leading ultimately to multinodular goiter. To determine the underlying mechanisms, we performed transcriptomic analyses on thyroid glands of mice at three age points: week 2 (W2, before visible growth retardation), W4 (at the nadir of growth); and W12 (immediately before full growth recovery). Using gene set enrichment analysis, we compared a defined set of thyroidal genes between Xb130^+/+^ and Xb130^−/−^ mice to identify differentially enriched gene clusters. At the earliest postnatal stage (W2), the thyroid glands of Xb130^−/−^ mice exhibited significantly downregulated gene clusters related to cellular metabolism, which continued to W4. Additionally, mutant thyroids at W4 and W12 showed upregulated gene clusters related to extracellular matrix, angiogenesis, and cell proliferation. At W12, despite nearly normal levels of serum TH and TSH and body size, a significantly large number of gene clusters related to inflammatory response were upregulated. Early postnatal TH deficiency may suppress cellular metabolism within the thyroid gland itself. Upregulation of genes related to extracellular matrix and angiogenesis may promote subsequent thyroid growth. Chronic inflammatory responses may contribute to the pathogenesis of multinodular goiter in later life. Some of the pathoadaptive responses of Xb130^−/−^ mice may overlap with those from other mutations causing congenital hypothyroidism.

## 1. Introduction

The thyroid gland synthesizes and releases thyroid hormone (TH), essential for promoting cellular metabolism, tissue function, and body growth. In congenital hypothyroidism, TH production by the thyroid gland is insufficient [1]. Causes of deficient TH synthesis include thyroid gland maldevelopment (dysgenesis), followed by genetic defects in TH synthesis [2,3,4]. Both thyroid gland development and the function of gene products involved in TH synthesis are critically dependent upon the development and maintenance of thyroid epithelial polarity.

Disruption of thyrocyte organization leads to various thyroid disorders [5,6]. XB130, also known as actin filament-associated protein 1-like 2 (AFAP1L2) [7], is predominantly expressed in the thyroid gland [7,8,9]. This adaptor protein, which localizes in the cytoplasm and is enriched at the apical plasma membrane, plays a crucial role in defining thyroid polarity and cytoskeletal architecture [10]. Specifically, global deletion of Xb130 in mice causes primary defects in thyroid gland development and function, including delayed folliculogenesis during embryonic and early postnatal stages, along with a reduction in thyroglobulin (Tg) release to the thyroid follicle lumen, with diminished iodination. XB130 knockout (Xb130^−/−^) mice exhibit transient postnatal growth retardation because of congenital hypothyroidism [11]. Interestingly, older Xb130^−/−^ mice develop multinodular goiter (MNG), characterized by an enlarged thyroid gland with focal degeneration resulting in nodule formation [12], indicating age-related response(s) to the genetic defects of the thyrocyte cytoskeleton, early thyroid gland development plus accompanying TH synthesis defects.

To understand these age-related compensatory changes, we performed microarray-based transcriptomic analysis of thyroid tissue collected at three different stages of postnatal development, accompanied by a bioinformatic analysis of enriched gene clusters [13,14].

## 2. Materials and Methods

### 2.1. Animals

Xb130^−/−^ mice were generated as described previously [15]. All procedures carried out in mice were approved by the Animal Use and Care Committee of the University Health Network (Toronto, ON, Canada). Mice were maintained in specific pathogen-free conditions on a 12 h light: 12 h dark cycle and fed an autoclaved laboratory chow and tap water ad libitum.

Thyroid tissue samples were collected from male mice at the ages of W2, W4, and W12. Following carbon dioxide inhalation, their thyroid glands were harvested under a surgical microscope (Leica M651, Leica Microsystems, Mannheim, Germany). Two groups (Xb130^+/+^ and Xb130^−/−^ mice) of thyroid tissue samples, consisting of four biological replicates, were prepared for each age point. The numbers of biological replicates were based on our previous experience in microarray studies with animals [16].

### 2.2. RNA Extraction and Microarray Analyses

Total RNA was extracted from the collected thyroid tissues using an RNeasy kit (Qiagen, Valencia, CA, USA). Equal amounts of RNA from each group were used for microarray. cDNA was synthesized using high-capacity cDNA reverse transcription kits (Applied Biosystems, Foster City, CA, USA). The RNA integrity number, determined by the Agilent Bioanalyzer 2100 (Agilent Technologies, Inc., Santa Clara, CA, USA), was used as a measure of the quality of the RNA. Mouse gene ST 2.0 chips (35,240 spotted genes) from Affymetrix (Santa Clara, CA, USA) were used.

Probe-level data were preprocessed, including background correction, normalization, and summarization, using robust multiarray average analysis. Data normalization was performed across all arrays using quantile normalization. The background-adjusted, normalized values were then compiled, or summarized, using the median polish technique, to generate a single measure of expression. Gene expression was centered and scaled to generate principal component analysis plots and heatmaps. Significant differentially expressed genes were selected at an FDR < 0.05. Original data are available from the Gene Expression Omnibus database (GSE197052).

### 2.3. Gene Set Enrichment Analysis

**Figure 1:**
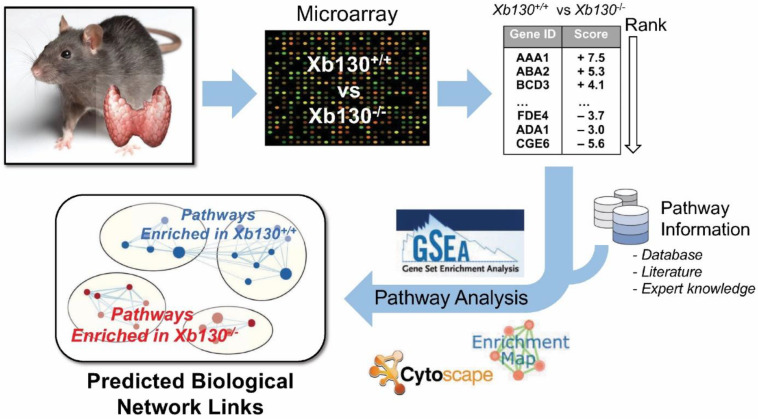
Transcriptomic profiling of gene expression in the thyroid glands of Xb130^−/−^ mice and their littermates. Gene set enrichment analysis is the major method that compares gene sets between the two different groups.

To create ranked lists for pathway analysis, genes were ranked based on a gene score. A pre-ranked gene set enrichment analysis (GSEA) was conducted on each ranked list [13]. Enriched pathways, which met the cut-off of false discovery rate (FDR) *p* < 0.001 and *p* < 0.005, were plotted together and clustered to group highly similar pathways using the EnrichmentMap and AutoAnnotate Cytoscape apps (Figure 1) [17,18].

## 3. Results

### 3.1. Transcriptomic Profiling of Thyroid Gland during Postnatal Development

**Figure 2:**
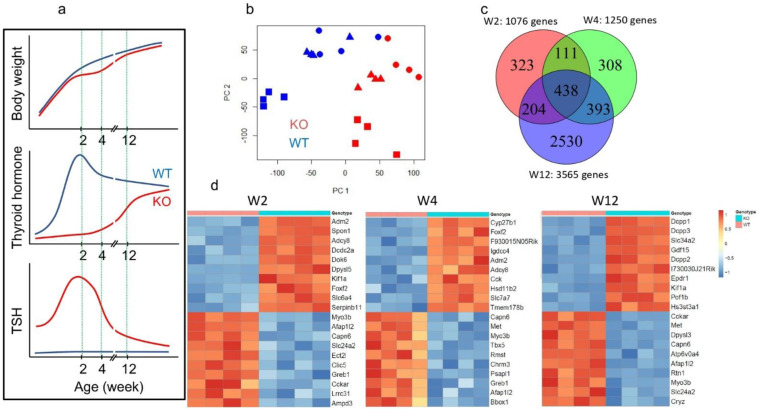
Differentially expressed genes in the thyroid glands between Xb130^+/+^ and Xb130^−/−^ mice (**a**) Transient growth retardation of Xb130^−/−^ mice due to congenital hypothyroidism and experimental design. See original data in [11]; (**b**) Principal Component Analysis (PCA) of all genes from each microarray shows distinct differences in gene expression profiles between the thyroid glands of Xb130^+/+^ and Xb130^−/−^ mice at each age point, with circles representing 2 weeks, triangles representing 4 weeks, and squares representing 12 months; (**c**) Venn diagram showing the overlap of differentially expressed genes (FDR < 0.05) among all 3 timepoints; (**d**) Heatmaps showing top 10 up- and down-regulated genes at each timepoint.

Xb130^−/−^ mice exhibit transient postnatal growth retardation with a nadir at around W4, which is fully recovered by W14 (Figure 2a, upper panel). In euthyroid Xb130^+/+^ mice, serum TH levels increase rapidly after birth and peak at W2, followed by a gradual decline to a relatively constant level around W14. In contrast, the serum TH levels in hypothyroid Xb130^−/−^ mice are exceedingly low, and gradually catch up with those of Xb130^+/+^ mice around W14 (Figure 2a, mid panel). As a result, Xb130^−/−^ mice have a dramatically higher level of serum TSH than Xb130^+/+^ mice, with a peak at W2 followed by a subsequent gradual decline, although it remains higher than that of Xb130^+/+^ mice through adulthood (Figure 2a, lower panel) (see detailed data [11]). To understand the adaptive responses of the thyroid gland to Xb130 deficiency and the consequent hypothyroidism, we performed microarray-based transcriptomics analyses on thyroid tissues collected at three age points: W2 (before visible growth retardation); W4 (at the nadir of growth); and W12 (immediately before full growth recovery).

**Figure 3:**
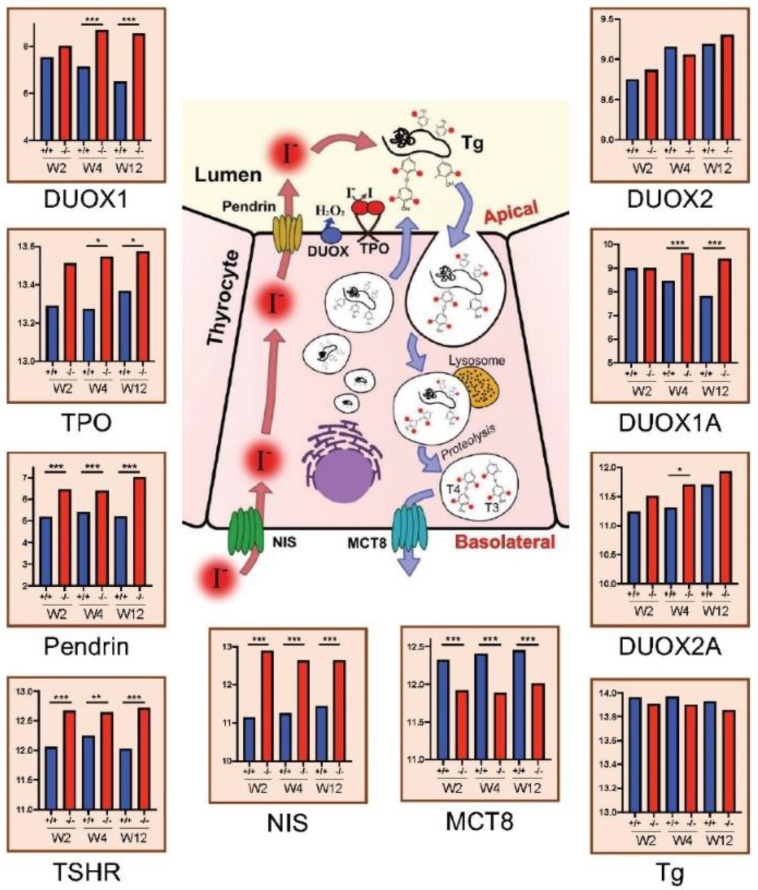
Gene expression of TH biosynthesis-related genes in thyroid glands at W2, W4 and W12 from microarray data (N = 4 in each group). Data are expressed as the Mean values. * *p* < 0.05, ** *p* < 0.01, *** *p* < 0.001.

Principal Component Analysis (PCA) shows distinct separation of overall gene expression profiles between thyroid glands of Xb130^−/−^ (Figure 2b; labeled in red) and Xb130^+/+^ mice (Figure 2b; labeled in blue), and among the three age points (labeled with different symbols), indicating that the deletion of Xb130 gene causes major gene expression changes in the mouse thyroid glands in an age-dependent manner (Figure 2b). The number of differentially expressed thyroidal genes between WT and KO increased from 1076 at W2 to 1250 at W4, and to 3565 at W12 (Figure 2c). The top up- and down-regulated genes showed distinct biological functions (Figure 2d). Genes related to TH biogenesis show a normal response to TSH stimulation in hypothyroidism, including Tpo, Pendrin, Tshr, Nis, Mct8, Duox1, Duox2, Duoxa1, and Duoxa2 (Figure 3). These genes were validated by RT-qPCR [11].

The dynamic changes in the gene-expression profiles were further demonstrated by GSEA [13]. Compared with thyroid glands from Xb130^+/+^ mice, those from Xb130^−/−^ mice show enrichment of eight downregulated and two upregulated gene clusters at W2, as well as six downregulated and four upregulated gene clusters at W4. The number of upregulated gene clusters increased to 47, with no downregulated clusters, at W12 (Table 1).

### 3.2. Downregulated Gene Clusters in Xb130^−/−^ Thyroid Glands at Early Postnatal Stages Are Related to Mitochondrial Energetics

**Figure 4:**
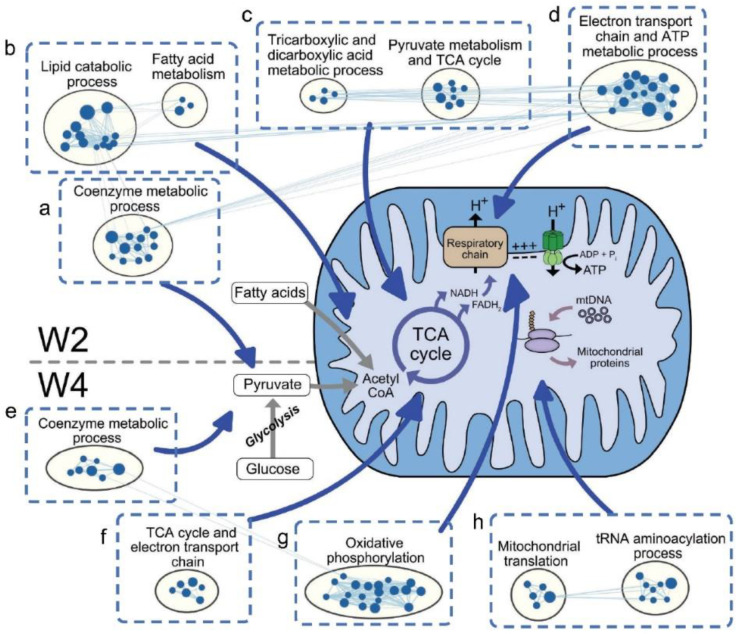
Downregulation of mitochondrial energetics-related gene clusters in thyroid glands of Xb130^−/−^ mice at W2 and W4. Enrichment results from GSEA were mapped as a network of gene sets. Blue nodes represent downregulated gene sets in Xb130^−/−^ mice.

Although the thyroid gland expresses TH receptors, there is a relative paucity of information about the impact of TH on the thyroid gland itself. Nevertheless, TH is a major endocrine regulator of metabolic rate, with a profound impact on mitochondria responsible for the majority of cellular ATP production, particularly on mitochondrial energetics and oxidative phosphorylation [19]. In fact, we found that most of the gene clusters downregulated at W2 and W4 in thyroid glands of Xb130^−/−^ mutant mice are related to mitochondrial energetics, including the coenzyme metabolic process (Figure 4a), as well as lipid catabolic process and fatty acid metabolism (Figure 4b), indicating reduced utilization of glucose and fatty acids for cellular metabolism. Downregulation of gene clusters regulating the TCA cycle and dicarboxylic acid metabolism, in addition to those related to pyruvate metabolism, was also observed (Figure 4c), as well as downregulation of the electron transport chain and ATP metabolic processes that affect respiration (Figure 4d).

As with W2, the thyroid glands of Xb130^−/−^ mice at W4 showed downregulated gene-clusters of the coenzyme metabolic process (Figure 4e), TCA cycle and electron transport chain (Figure 4f), and oxidative phosphorylation (Figure 4g), all of which favors reduced cellular metabolism in the thyroid gland. Additionally, gene clusters of tRNA aminoacylation and mitochondrial translation processes were downregulated (Figure 4h). As these processes are all associated with the biogenesis of mitochondria and mitochondrial mass, they seem compatible with the reported actions of hypothyroidism in other thyroid hormone target tissues.

### 3.3. Upregulation of Gene Clusters Related to Tissue Development in Xb130^−/−^ Thyroid Glands at W4 and W12

**Figure 5:**
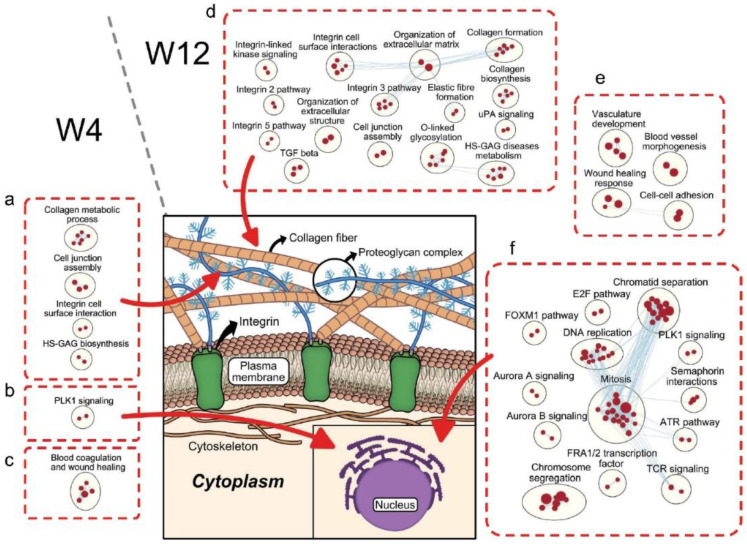
Upregulation of tissue development-related pathways in the thyroid glands of Xb130^−/−^ mice at W4 and W12. Pathways associated with extracellular matrix at W4 (**a**) and W12 (**d**). Pathways associated with cell cycle regulation at W4 (**b**) and W12 (**f**). Pathways associated with angiogenesis, blood coagulation and wound healing at W4 (**c**) and W12 (**e**). Red nodes represent upregulated gene sets in Xb130^−/−^ mice.

While downregulation of gene-clusters related to mitochondrial energetics in the thyroid glands of Xb130^−/−^ mice tend to resolve in parallel with an increase in circulating TH as a function of age, interestingly, other gene clusters become progressively upregulated through development (Figure 5). At W4, processes associated with extracellular matrix (ECM), including collagen metabolic process, cell junction assembly, integrin–cell-surface interactions, and heparan sulfate/glycosaminoglycan (HS-GAG) biosynthesis, begin to become upregulated in the thyroid glands of Xb130^−/−^ mice (Figure 5a). Moreover, the thyroid glands exhibit a small number of upregulated gene clusters related to maturation of centrosomes (PLK 1 pathway) (Figure 5b) [20]. Given the relationship of centrosomes as the microtubule organizing center, and the critical role of cell junctions as well as cell–ECM interactions in epithelial function, these responses appear to represent successful thyroidal compensation to overcome the defective epithelial polarity brought about by loss of XB130. The pathway known as blood coagulation and wound healing (Figure 5c) also appears to be linked to ECM structure and function.

Noticeably, upregulation of these processes continued to be further elevated at W12, including organization of ECM (collagen formation and biosynthesis), integrin function (integrin-linked kinase, integrin–cell-surface interactions, integrin 2, 3, and 5 pathways), HS-GAG, O-linked glycosylation, and cell junction assembly (Figure 5d). Gene clusters related to wound healing, and cell–cell adhesion were also upregulated (Figure 5e).

Consistent with our previous observation that the thyroid glands of Xb130^−/−^ mice exhibit elevated cell proliferation and growth at W4 and W14 [11], gene clusters related to cell cycle regulation, such as chromatid separation, DNA replication, chromosome segregation, and mitosis, became highly upregulated at W12 (Figure 5f). Upregulation of processes related to cell-cycle regulations (FOXM1, E2F, FRA1/2, ATR) and maturation of centrosomes (PLK1, Aurora A, Aurora B) were also observed. Interestingly, two gene-clusters related to immune function (semaphorin interactions and TCR) showed interactions with mitosis-related gene clusters (Figure 5f). Further, gene clusters in the thyroid glands of Xb130^−/−^ mice supporting angiogenesis (vasculature development, blood vessel morphogenesis), as well as cell proliferation, likely contribute to compensatory enlargement of the entire thyroid gland (i.e., a goiter comprised not only of thyrocytes but also of blood supply) stimulated by TSH, leading to increased TH production.

### 3.4. Elevated Inflammatory Response-Related Pathways in Xb130^−/−^ Thyroid Glands at W12

**Figure 6:**
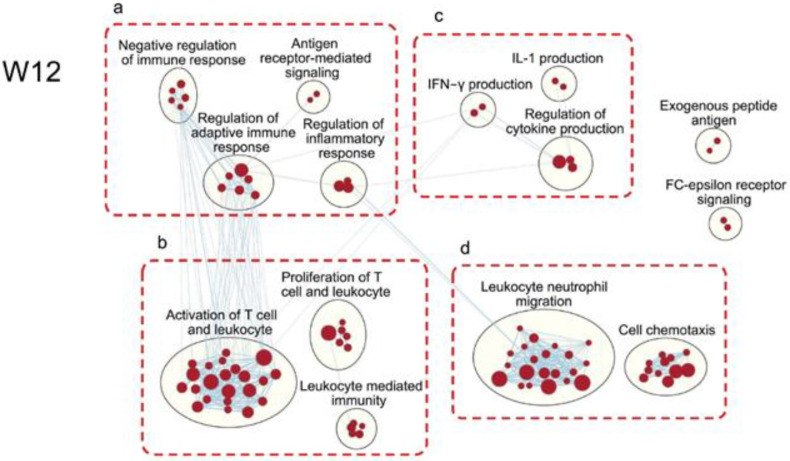
Upregulation of immune response-related pathways in the thyroid of Xb130^−/−^ mice at W12.

Interestingly, many gene clusters related to inflammatory responses were found to be upregulated in the thyroid glands of Xb130^−/−^ mice at W12 (Figure 6), a time when the serum TH and TSH levels in the circulation are nearly normal (Figure 2a). These included negative regulation of immune response, antigen receptor-mediated signaling, regulation of adaptive immune response, and regulation of inflammatory response (Figure 6a), in addition to elevated activation and proliferation of T cells and leukocytes (Figure 6b). Our analysis also showed upregulation of gene clusters related to the regulation of cytokine production, especially interferon-gamma and IL-1 (Figure 6c). Furthermore, processes of leukocyte and neutrophil migration and cell chemotaxis were upregulated (Figure 6d). These results indicate that even though the hypothyroidism in Xb130^−/−^ mice is almost fully recovered by W12 through TSH-related compensatory growth of the thyroid gland, the gland remains abnormal, as chronic inflammation persists, which may contribute to the MNG formation at later stages of life [12].

## 4. Discussion

In this study, our transcriptomic analyses revealed dynamic changes in the thyroid glands of mice adapting to Xb130 deficiency. At early postnatal stages, while congenital hypothyroidism delays the growth of the whole body, the thyroid gland suffers from impaired cellular metabolism that seems to suggest the effect of insufficient TH on the thyroid gland itself. Subsequently, after the nadir of growth, the thyroid gland begins to enlarge under TSH stimulation with upregulated genes related to ECM, cell proliferation and angiogenesis. These data support recent studies demonstrating that thyroid gland growth is needed to help to alleviate TH deficiency in congenital hypothyroidism [21], and this can eventually help to reverse the reduction in cellular metabolism in the thyroid gland and throughout the body.

### 4.1. Effects of TH Deficiency on Cellular Metabolism in the Thyroid Glands at Early Postnatal Stages

Many studies thus far have demonstrated the metabolic effects of TH on virtually all organs in the body, including the heart, brain, and liver [22], but reports of the metabolic consequences of TH deficiency in the thyroid gland itself are scarce. The present study suggests that the reduced levels of TH production that are known to downregulate genes related to cell metabolism in other hypothyroid cell and tissue types [23,24] also bring about similar effects in the thyroid gland. Specifically, our analyses revealed downregulation of gene clusters associated with glycolysis, fatty acid and lipid metabolism, TCA cycle, and oxidative phosphorylation in the thyroid glands of Xb130^−/−^ mice at W2 and W4 (Figure 4a–g). In addition, decreased pathways of mitochondrial protein synthesis observed in their thyroid glands at W4 (Figure 4h) indicate that not only cellular respiratory capacity but also mitochondrial biogenesis is reduced in Xb130^−/−^ thyroid glands, which is consistent with reports that TH acts directly on mitochondrial protein synthesis [25,26]. Thus, given the inability of Xb130^−/−^ thyroid glands to produce enough TH for growth (Figure 2a) [11], these observations seem quite likely to reflect the consequences of TH deficiency on the thyroid gland itself.

### 4.2. Compensation for Defects in Thyroid Epithelial Polarity

Congenital hypothyroidism with dysgenesis/dyshormonogenesis may be caused by a number of monogenic defects, to which XB130 is a recently identified member [10,11]. Lack of XB130 in thyrocytes interferes with the normal crosstalk between the microtubule network and the actin cytoskeleton, leading to disturbances in thyroid epithelial polarity [10]. Thus, lack of XB130 is one of the few single gene mutations so far identified that is genetically linked to both a thyroid developmental defect (dysgenesis) and deficiency of thyroid function, as detected by diminished Tg secretion and iodination (dyshormonogenesis). We hypothesize that some of the compensation in Xb130^−/−^ mice represents a specific adaptive response to the perturbation of epithelial polarity in the thyroid gland. With this in mind, we note transcriptomic upregulation associated with ECM as early as postnatal W4 and strengthening with age, including centrosomal maturation, collagen formation and biosynthesis, integrin–cell-surface interactions, HS-GAG biosynthesis, O-linked glycosylation, cell junction assembly, and cell–cell adhesion (Figure 5). Each of these pathways appear to be part of the successful compensation of thyroid tissue architecture [27] in the setting of defective epithelial polarity brought about by loss of XB130.

### 4.3. Thyroid Gland Growth and Remodeling

The enlargement of the thyroid gland, or goiter, is an established compensatory mechanism for defective TH production, and it often occurs gradually over time. We have previously reported on compensatory growth of thyroid glands in Xb130^−/−^ mice [11], and our transcriptomic analyses of Xb130^−/−^ thyroid glands show the emergence of tissue development-related pathways at W4 (including activation of cell-cycle regulation and angiogenesis (Figure 5a–c)) that are only further enriched by W12 (Figure 5d–f). TH deficiency is known to trigger the production of TSH in the pituitary, and TSH stimulates the compensatory growth of the thyroid gland with an increase in cell proliferation. The intracellular signaling of TSH through its receptor is mediated through the cAMP pathway. XB130 is involved in cAMP-dependent potentiation of IGF-1-induced DNA synthesis in rat FRTL-5 thyroid cells [28]. Angiogenesis is essential for all tissue growth and occurs in the thyroid under goitrogenic stimulation [29,30,31]. Presumably, the stimulatory activity of TSH receptors occurs primarily on thyrocytes, which may themselves secrete factors that stimulate angiogenesis locally. Further investigation of these processes is needed to understand the underlying molecular and cellular mechanisms of compensatory enlargement and remodeling in the thyroid glands under hypothyroidism.

### 4.4. Early Hypothyroid-Triggered Inflammatory Responses in Thyroid Glands

One of the most interesting and unexpected findings in the present study is the overwhelming number of differentially regulated genes (Figure 2c) and enrichment of gene-clusters related to inflammation in the thyroid glands from Xb130^−/−^ mice at W12 (Figure 6), at an age when their serum TH and TSH levels approach closer to euthyroidism (Figure 2a) [11]. Although enlarged thyroid follicles were observed in Xb130^−/−^ mouse thyroid glands at this gestational stage using morphometric analysis, we found no obvious infiltration of leukocytes and lymphocytes in the thyroid tissue [11]. However, their thyroid glands at this age were found to have elevated expression of genes related to inflammatory response, featured by upregulated gene clusters in activation of T cells and leukocytes and neutrophil migration, which may contribute to the pathogenesis of MNG observed in aged Xb130^−/−^ mice [12].

The immune system plays a central role in tissue repair and remodeling. Upon wound injury, inflammation attempts to repair tissue damage and to restore tissue homeostasis by recruiting various immune cells to the site of injury [32,33], but chronic or uncontrolled inflammation can lead to pathological tissue remodeling or fibrosis [34]. Thus, the activation and elevation of the inflammatory response in the thyroid glands of Xb130^−/−^ mice, occurring concomitantly with enrichment of genes related to tissue remodeling (Figure 5), appears to be a pathoadaptive response. This raises important questions for future study of the underlying molecular triggers for the enhanced inflammatory response that has been observed as a common pathological characteristic exhibited in patients with thyroid disorders [35]. Despite many years of work internationally, the pathogenesis of MNG remains ill-defined. As in many disease states, there are likely to be both environmental and genetic influences. One long-term consequence of inflammation can be increased local fibrosis, and this is observed histologically in many individuals with MNG.

Even though XB130 is highly expressed in the thyroid gland [7,8,9], it is also moderately expressed in other organs. The lack of XB130 enhances lipopolysaccharide-induced septic responses and acute lung injury [36], and carcinogen-induced skin tumorigenesis that is mediated through chronic inflammation [37]. The role of XB130 in mediating inflammatory responses should be further investigated.

### 4.5. Limitations of the Present Study

In the present study, we only studied transcriptomic profiles in male Xb130^+/+^ and Xb130^−/−^ mice, as the transient postnatal growth retardation was found in both male and female Xb130^−/−^ mice [11]. We also did not see differences in MNG formation between male and female mice [12]. However, in carcinogen-induced skin tumors, the numbers of tumors were increased only in male Xb130^−/−^ mice [37]. The growth of Xb130^+/−^ mice was compatible with WT mice in both males and females [11]. However, even though the incidence of MNG in Xb130^+/−^ mice is very low, it is statistically higher than that in Xb130^−/−^ mice [12]. Moreover, in carcinogen-induced skin tumorigenesis, male Xb130^+/−^ mice showed similar increased tumor numbers to that in Xb130^−/−^ mice [37]. The effects of sex and “gene dose” could be dependent on different clinical situations and should be further studied. Furthermore, the results from the bioinformatics study should be validated with functional studies.

## 5. Conclusions

In the present study, we exploited congenitally hypothyroid Xb130^−/−^ mice to visualize dynamic changes in gene expression that occur in their thyroid gland through postnatal stages. The transcriptomic profiles observed in this study may be shared with those of thyroid dysfunction caused by other genetic mutations or functional defects. This deserves further investigation in other animal models and in humans, as such studies may lead to the discovery of pathways that promote the development of thyroid disease in adulthood, such as multinodular goiter.

## Figures and Tables

**Table 1:** Differentially expressed gene-set clusters in thyroid glands between Xb130^+/+^ and Xb130^−/−^ mice. Gene-set clusters enriched in Xb130^+/+^ group. Gene-set clusters enriched in Xb130^−/−^ group.

**Week 2**		
Lipid catabolic process	Fatty acid metabolism	Coenzyme metabolic process
Electron transport chain and ATP metabolic process	Tricarboxylic and dicarboxylic acid metabolic process	Pyruvate metabolism and TCA cycle
Cellular response to interferon-beta	Triglyceride metabolic process	
Signal unattached Mad2	Generation messenger molecules	
**Week 4**		
Coenzyme metabolic process	Oxidative phosphorylation	TCA cycle and electron transport chain
Mitochondrial translation	tRNA aminoacylation process	Intracellular transmembrane protein
Collagen metabolic process	Cell junction assembly	Integrin–cell-surface interaction
HS-GAG biosynthesis	PLK1 signaling	Blood coagulation and wound healing
Negative adaptive immune		
**Week 12**		
Negative regulation of immune response	Antigen receptor-mediated signaling	Regulation of adaptive immune response
Regulation of inflammatory response	Activation of T cell and leukocyte	Proliferation of T cell and leukocyte
Leukocyte mediated immunity	IFN-γ production	IL-1 production
Regulation of cytokine production	Leukocyte neutrophil migration	Cell chemotaxis
Exogenous peptide antigen	FC-epsilon receptor signaling	Integrin-linked kinase signaling
Integrin–cell-surface interactions	Integrin 2 pathway	Organization of extracellular structure
Integrin 5 pathway	TGF beta	Organization of extracellular matrix
Integrin 3 pathway	Cell junction assembly	Elastic fiber formation
O-linked glycosylation	Collagen formation	Collagen biosynthesis
uPA signaling	HS-GAG diseases metabolism	Vasculature development
Blood vessel morphogenesis	Wound healing response	Cell–cell adhesion
FOXM1 pathway	Aurora A signaling	Aurora B signaling
Chromosome segregation	E2F pathway	DNA replication
Mitosis	FRA1/2 transcription factor	Chromatid separation
PLK1 signaling	Semaphorin interactions	ATR pathway
TCR signaling		

## Data Availability

Some or all data generated or analyzed during this study are included in this published article or in the data repositories listed in References.
